# Evaluation of registered nurses’ interprofessional emergency care competence through the gamification of cardiopulmonary resuscitation training: a cross-sectional study

**DOI:** 10.1186/s12909-023-04332-y

**Published:** 2023-05-22

**Authors:** Tzu-Sang Chen, Pei-Lun Hsieh, Chien Chien Tung, Chao-Hsin Wu, Yu-Chieh Cheng

**Affiliations:** 1 Department of Nursing, Tungs’ Taichung MetroHarbor Hospital, Taichung, Taiwan R.O.C.; 2 Department of Nursing, College of Health, National Taichung University of Science and Technology, Taichung, Taiwan R.O.C.; 3 Jie-Rui Social Welfare Foundation Elderly Care Center, Nantou, Taiwan R.O.C.; 4 Department of Emergency Medicine, Tungs’ Taichung MetroHarbor Hospital, Taichung, Taiwan R.O.C.; 5 Department of Othopedics, Tungs’ Taichung MetroHarbor Hospital, Taichung, Taiwan R.O.C.

**Keywords:** Gamification, Game-based learning, Emergency care, Nursing competence

## Abstract

**Backgrounds:**

Cardiopulmonary resuscitation (CPR) training is generally led by instructors in a classroom; thus, conventional teaching materials used in CPR training are often constrained by spatiotemporal factors, limiting learners’ interest and sense of achievement in learning and preventing them from effectively applying what they learn in practice. For greater effectiveness and more flexible application, clinical nursing education has increasingly emphasized contextualization, individualization, and interprofessional learning. This study determined the self-assessed emergency care competencies of nurses who received gamified emergency care training and explored the factors associated with those competencies.

**Methods:**

Quota sampling of nurses working at a certain regional hospital in central Taiwan was conducted, and a structured questionnaire was administered to the recruited nurses. A total of 194 valid responses were collected. The research tool was a scale measuring the participants’ emergency care competencies after they received gamified emergency care training. The data were analyzed using descriptive and inferential statistics and multiple regression.

**Results:**

Of the recruited participants, 50.52% were ≤ 30 years old; 48.45% worked in the internal medicine department; 54.64% graduated from 2-year university technical programs; 54.12% were N2 registered nurses; 35.57% and 21.13% had ≥ 10 and 1–3 years of work experience, respectively; and 48.45% worked in general wards. User need (*r* = 0.52, *p* = 0.000), perceived usefulness (*r* = 0.54, *p* = 0.000), perceived ease of use (*r* = 0.51, *p* = 0.000), and usage attitude (*r* = 0.41, *p* = 0.000) were positively correlated with emergency care competencies. Furthermore, the multiple regression analysis revealed that perceived usefulness was the primary factor associated with the participants’ emergency care competencies.

**Conclusions:**

The results of this study may serve as a reference for acute care facility authorities in designing advanced nursing competency standards and emergency care training programs for nurses.

## Introduction

Technological advancements have transformed interprofessional health-care education. Innovative teaching strategies, in which newly developed technologies are applied in clinical care nursing, have been recently employed in health-care education [1]. Digital learning—including massive open online courses, flipped classrooms, ubiquitous learning, virtual learning environments, and, since 2017, game-based learning (GBL)—has been applied in medical and nursing education [1–3]. In recent years, the quality of clinical medical education has been improved through contextualization, individualization, gamification, and the incorporation of action learning [4, 5].

Digital technologies have been broadly applied in medical education curricula, and simulation-based clinical teaching worldwide [6, 7]. Studies have reported the benefits of digital technology in the education of medical students and have explored the applications of information technology in medicine, including its applications in interactive teaching using 3D anatomical images, nursing simulations, and objective structured clinical examinations [3–5, 7].

Nurses are eyewitnesses of and first-aid providers during patient emergencies and are often the first professionals on a health-care team to assess a patient’s need for first aid at the scene [3, 8]. According to the European Research Council, emergency care competencies are core skills for nurses, including sufficient knowledge of first-aid and cardiopulmonary resuscitation (CPR) techniques, assessment of emergencies and proper administration of first-aid procedures, maintenance of airway and recovery position, knowledge and recognition of timely and effective breathing, accurate assessment of vital signs, cardiac massage with appropriate speed and pressure, and appropriate use of automatic external defibrillators [9]. Conventional emergency care training has been limited by spatial and temporal factors, limiting learners’ interest and sense of achievement in learning and preventing learners from applying what they learn in practice [1, 3, 4]. Recently, GBL has emerged as a potential solution to this problem; however, further research on its effectiveness in improving the professional competencies of health-care providers is needed [10, 11].

As a matter of fact, CPR is a common clinical scenario and a mandatory competency for medical professionals [3, 8]. According to Taiwan’s Emergency Medical Services Act, health-care professionals working in emergency and intensive care units must possess advanced cardiac life support (ACLS) certificates [3, 12, 13]. Hsieh et al. [12] concluded that both situational simulation training and online teaching were effective in improving the first-aid competencies of nurses in general medical wards.

Since the dawn of the Internet age, smart devices have become increasingly prevalent in people’s daily lives. Many educators have adopted innovating teaching strategies involving the use of new technologies and flipped-classroom models. Furthermore, GBL has been applied in various medical education contexts [5]. Venkatesh et al. [14] proposed the Unified Theory of Acceptance and Use of Technology (UTAUT) model, which aims to measure users’ intentions to use an information system and their subsequent usage behaviors, summarized as the dimensions of perceived usefulness (i.e., users’ perceptions regarding the usefulness of learning for improving their competence and confidence and preventing them from making mistakes at work) and perceived ease of use (i.e., users’ perceptions that a learning platform or system motivates them to learn, has a clear interface, and is easy to use).

Conventional teaching models involve one-way delivery of knowledge to learners. However, successful learning requires learners to be motivated to learn. Insufficient learning content undermines learners’ attention and interest, resulting in less favorable learning outcomes. GBL combines learning content with games to inspire learners to learn continually through practice and repetition, thus enhancing their learning outcomes [9, 10, 15, 16].

GBL has been explored in medical education research. Gallegos et al. [17] applied the 3D Gamelab gaming platform in a preliminary nursing education course in which 120 university nursing students participated. Each session lasted for 30–60 min and involved the use of handouts, videos, learning activities, and links to resources. After the students completed the learning activities, they were awarded badges. Qualitative interviews were conducted with the students to assess their experiences with using the learning platform and revealed that GBL inspired them to proactively participate in the course and maximize their learning potential.

GBL involves providing learners with a rich learning medium and an environment that stimulates their learning motivation. Games must be designed in consideration of elements such as the users’ knowledge, skills, and sensory stimulation; art design; rewards, characters; and soundtracks as well as the learners’ affective, behavioral, cognitive, and social or cultural engagement to motivate learners to accomplish their learning goals [8–10, 16]. This study investigated the self-assessed emergency care competencies of nurses who received gamified emergency care training and explored the factors associated with those competencies.

## Methodology

### Study design

This study adopted a cross-sectional design employing the UTAUT [14] and GBL [10] as its theoretical basis. A structured questionnaire was developed to assess hospital nurses’ acceptance of the emergency care game software and their nursing competencies after completing an on-the-job training course based on the gamification software.

Gamification involves using game attribute categories outside the context of a game that can stimulate learning-related behaviors or attitudes. The gaming software product was designed based on Landers’ gamified learning theory [18] and by condensing the ACLS guidelines [11]. This software product has acquired a utility model patent (Patent Number: M637730, applied on 2022/10/28).

By providing nurses with a fun, convenient, and repeatable memory-enhancing method not limited by space, the gamified CPR care scenario facilitates the acquisition of CPR knowledge and skills. The game design involves a combination of simulated situations, game interface variations, and reminder functions. The game content is presented through challenge themes, time-limited answers, and self-competition to guide players and enhance their learning motivation and operational intent.

**Fig. 1:**
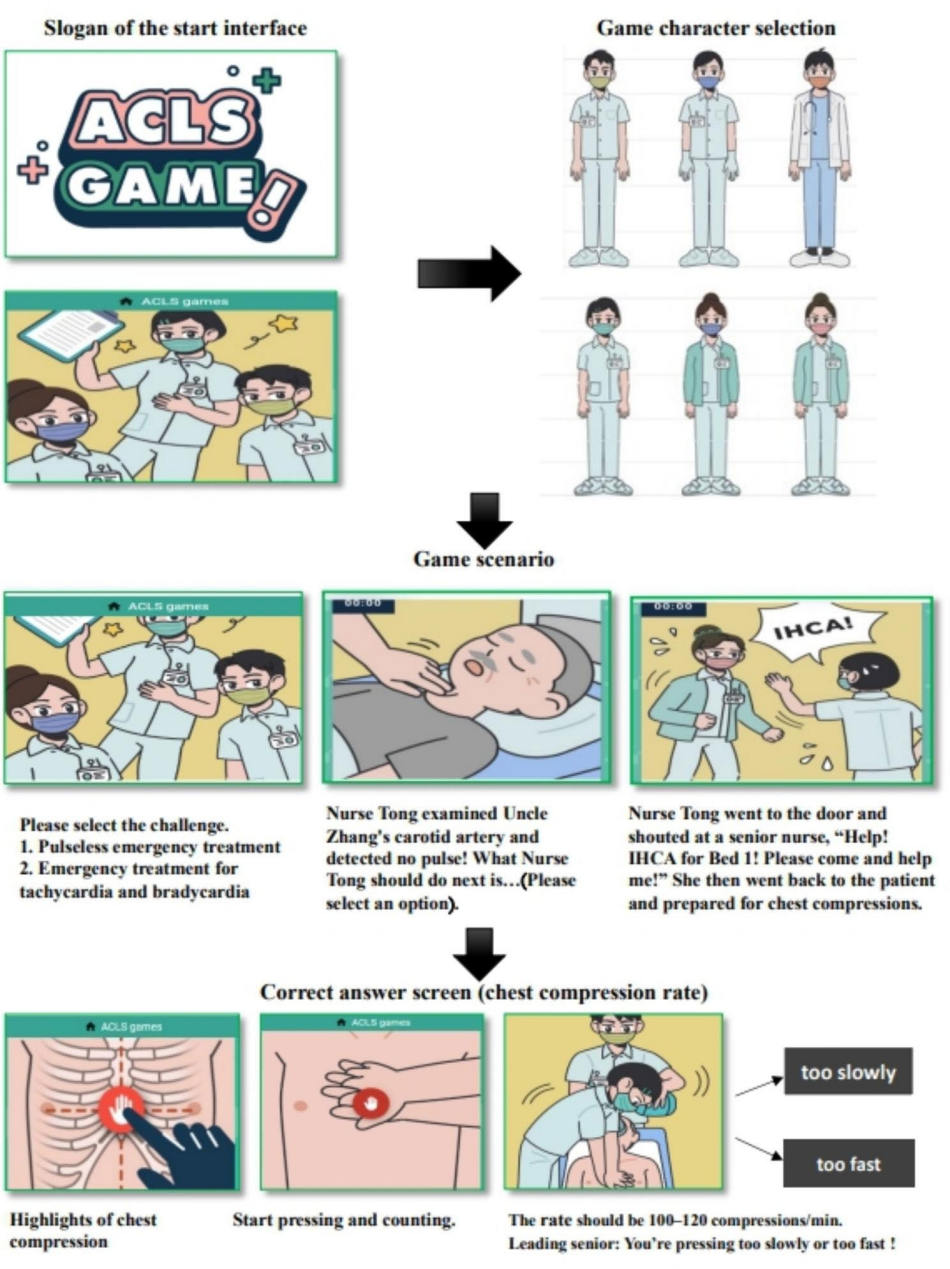
Introduction to the game process, character and scenario

**Fig. 2:**
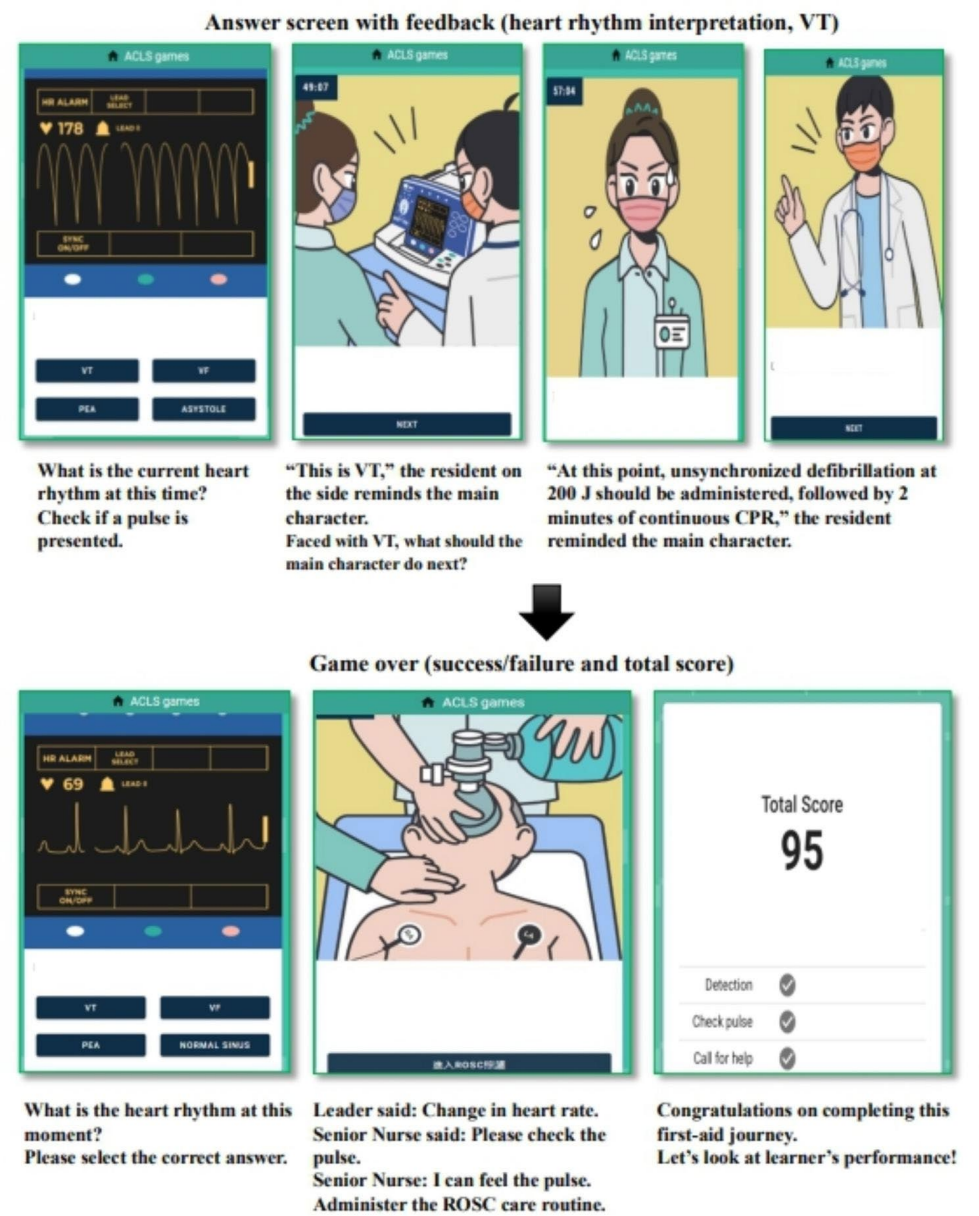
Game answer screen and total score with feedback

Regarding the game operation process (Fig. 1), the player enters the game website (https://tung-acls-public.web.app/) through a cell phone or computer, plays the game as the main character, spends one day in the game as a nurse taking care of patients, and passes the red, yellow, and blue zones (in that order). Each of these zones has its own challenge themes and predetermined decision points. At each decision point, the user can choose an option by tapping within 110 s; the game ends if the player takes longer. Completing all the tasks associated with each theme within the given time frame yields a full score of 100. The score is revealed at the end of the game. This zone has six simulated decision points, namely ventricular tachycardia (VT), ventricular fibrillation, pulseless electrical activity, asystole and other heart rhythms, chest compression location, and defibrillation; these points are presented randomly to train the player in identifying and treating abnormal heart rhythms. The game ends and provides feedback automatically if the player does not pass the challenge within 15 s or chooses the wrong path (Fig. 2).

### Study population

The participants in this study were nurses who had worked for at least 3 months at a certain regional hospital in central Taiwan and completed the program based on the emergency care game developed in 2021, whose software application and website was also provided to all the nurses (learners) in the hospital.

### Sample size

Determination of the sample size was based on Krejcie and Morgan’s [19] statistical tool, which provides a sample size for a finite population at a 95% level of confidence. The sample size of 150 was deemed representative of the estimated nursing population of approximately 1500 in the hospital, under consideration of a 5% nonresponse rate. Quota sampling was conducted according to the seniority of each ward nurse. In consideration of sample attrition, the questionnaire was administered to 200 participants recruited between January and March 2022; among these participants, 26, 40, 33, 26, and 75 had worked for 3 months to 1 year, 1–3 years, 4–6 years, 7–9 years, and ≥ 10 years, respectively.

### Research instrument

The questionnaire was self-developed and designed according to the goals and structure of this study. It consisted of three parts that respectively collected information on the nurses’ demographic backgrounds (13 items), acceptance of the game-based emergency care training program (23 items, revised UTAUT model [14] questionnaire), and perception of emergency care competencies (27 items). The third part involved the 27-item Nurses’ Aid Competency Scale self-developed based on the European Research Council [11] and Hsieh et al. [12]. Details of these three parts are presented as follows:

1. Demographic information: self-reported details on the demographic backgrounds of each participant, including their gender, age, seniority, and experience with using game-based/technological products.
2. Acceptance of the game-based emergency care training program: examines the participants’ acceptance of the game-based emergency care learning aids game software, including the user need, perceived usefulness, perceived ease of use, and usage attitudes toward the learning aids. Scored on a 5-point Likert scale (*“strongly agree,” “somewhat agree,” “neither agree not disagree,” “somewhat disagree,” and “strongly disagree”*).
3. Self-assessed emergency care competencies: assesses the participants’ clinical nursing competencies after they completed the game-based emergency care training. Each item was scored on a 5-point Likert scale (*“strongly agree,” “somewhat agree,” “neither agree not disagree,” “somewhat disagree,” and “strongly disagree”*).

### Questionnaire validity and reliability

The content validity of the questionnaire was assessed by experts, namely two doctors, one nurse, and two nursing professors. According to the content validity index calculation method proposed by Polit and Beck [20], the items that scored higher than 3 points were retained. The content validity indices of the scales on the acceptance of the game-based emergency care training program and the participants’ self-assessed emergency care competencies were 0.7 and 1, respectively, indicating that the content validity of both scales was satisfactory. The Cronbach’s α values of the aforementioned scales on the participants’ acceptance and competencies were 0.95 and 0.99, respectively, indicating that the internal consistency of both scales was satisfactory.

### Data collection

The participants were selected from the nurses that met the inclusion criteria, received explanations of the goal and procedure of this study, and signed written informed consent documents before their data were collected. The completed questionnaires were submitted directly to the researchers in sealed envelopes from February 2, 2022, to March 20, 2022. The participants were permitted to decline answering some of the items or withdraw from the study without consequences.

### Data analysis

SPSS 26.0 was used to analyze the collected data. A descriptive statistical analysis was conducted to evaluate the distributions of the percentages, means, and standard deviations in the data. Data were analyzed using a one-way analysis of variance (ANOVA) with the following independent variables: age, educational level, registered nurse level, years of seniority at the hospital, workplace unit type, and frequency of first-aid administration. In addition, correlation analyses were performed to explore the association between acceptance of the GBL training and emergency care competencies. A *p* value of < 0.05 was considered statistically significant.

## Results

From the 200 invitations to participate, 194 valid responses were obtained (response rate: 97%); six questionnaires were removed because they were almost entirely incomplete. Of the participants, 97.94% were female; 50.52% were ≤ 30 years old; 54.64% had graduated from a 2-year technical program; 54.12% were N2 registered nurse level; and 35.57% and 21.13% had provided clinical services for ≥ 10 and 1–3 years, respectively. Regarding the types of units in which the participants worked, 48.45%, 13.92%, 13.40%, and 24.22% worked in general wards, operating rooms, outpatient clinics, and other types of units, respectively. Among the participants, 90.21% and 7.22% administered CPR 1–2 times per year and 1–2 times per month, respectively (Table 1).

**Table 1:** Demographic information of the participants (*N* = 194)

	*N*	%	Cumulative %
**Sex**			
Female	190	97.94	97.94
Male	4	2.06	100.00
**Age (years)**			
≤ 30	98	50.52	50.52
31–40	60	30.93	81.44
41–50	34	17.53	98.97
≥ 51	2	1.03	100.00
**Educational level**			
Junior college (5-year program)	41	21.13	21.13
University (2-year technical program)	106	54.64	75.77
University (4-year technical program)	37	19.07	94.85
General university	5	2.58	97.42
Research institute	5	2.58	100.00
Doctoral program	0	0.00	100.00
**Registered nurse level**			
N0–N1	75	38.66	38.66
N2	105	54.12	92.78
N3	14	7.22	100.00
N4	0	0.00	100.00
**Seniority at the hospital (years)**			
< 1	27	13.92	13.92
1–3	41	21.13	35.05
4–6	28	14.43	49.48
7–9	29	14.95	64.43
≥ 10	69	35.57	100.00
**Unit type**			
Outpatient	26	13.40	13.40
General ward	94	48.45	61.86
Operating room	27	13.92	75.78
Other	47	24.22	100.00
**First-aid administration frequency**			
Once per day	1	0.52	0.52
1–2 times per week	4	2.06	2.58
1–2 times per month	14	7.22	9.79
1–2 times per year	175	90.21	100.00

The participants scored between 4.18 and 4.46 points on the self-assessed emergency care competency scale. They scored the highest and lowest on “first-aid assessment and initial treatment” and “arrhythmia assessment and treatment,” respectively. They scored between 3.53 and 4.42 points on the usage attitude scale, scoring the highest and lowest on the usage attitudes components, respectively. The participants’ average of user need, perceived usefulness, and perceived ease of use scores were 4.34, 4.30, and 4.36 points, respectively (Table 2).

**Table 2:** Participants’ (*N* = 194) acceptance of the game-based emergency care training program and self-assessed emergency care competencies

Dimension	Subdimension	Mean	SD
Acceptance of the game-based training program (13 items)	User need	4.34	0.62
	Perceived usefulness	4.30	0.66
	Perceived ease of use	4.36	0.62
Usage attitude (10 items)	Cognitive	4.42	0.58
	Affective	3.53	0.69
Emergency care competencies (27 items)	Maintenance of respiratory tract and circulation	4.30	0.70
	Teamwork and exception handling	4.26	0.62
	Arrhythmia assessment and treatment	4.18	0.64
	First aid assessment and initial treatment	4.46	0.59
	Nursing and emergency care performance	4.21	0.65

Correlations between pairs of variables for which the *r* coefficient was ≥ 0.7 (*p* < 0.05), 0.5–0.69 (*p* < 0.05), 0.30–0.49 (*p* < 0.05), and ≤ 0.29 (*p* < 0.05) were considered strong, moderately strong, moderate, and weak, respectively (Table 3). The participants’ emergency care competency scores were positively correlated with their user need (*r* = 0.52, *p* = 0.000), perceived usefulness (*r* = 0.54, *p* = 0.000), perceived ease of use (*r* = 0.51, *p* = 0.000), and usage attitude (*r* = 0.41, *p* = 0.000) scores (Table 3**)**.

**Table 3:** Correlation coefficients of emergency care competencies

Variable	User need	Perceived usefulness	Perceived ease of use	Usage attitude	Emergency care competencies
User need	1				
Perceived usefulness	0.80***	1			
Perceived ease of use	0.76***	0.78***	1		
Usage attitude	0.58***	0.63***	0.66***	1	
Emergency care competencies	0.52***	0.54***	0.51***	0.41***	1

*p < 0.05; **p < 0.01; ***p < 0.001

The predictive power of the predictor variables (i.e., user need, perceived usefulness, perceived ease of use, and usage attitude) for the dependent variable (i.e., emergency care competencies) was evaluated through a multiple regression analysis. According to the ANOVA results, the overall effect in the regression model reached statistical significance (*F* = 22.25, *p* = 0.00 < 0.05). The adjusted coefficient of determination (0.31) indicated that the model’s overall explanatory power was 30.6%.

Although the *F* values of the regression model reached statistical significance, the standardized regression coefficients of user need (*β* = 0.17, *t* = 1.59, *p* = 0.11 > 0.05), perceived ease of use (*β* = 0.15, *t* = 1.41, *p* = 0.16 > 0.05), and usage attitude (*β* = 0.04, *t* = 0.53, *p* = 0.60 > 0.05) did not reach statistical significance. Perceived usefulness (*β* = 0.25, *t* = 2.25, *p* = 0.03 < 0.05) significantly affected the participants’ emergency care competencies (Table 4**)**.

**Table 4:** Regression analysis of the effects of predictor variables on emergency care competencies

Model	Nonstandardized coefficients		Standardized coefficients	*t*	Sig(*t*)	Adjusted *R*^2^		*F*	Sig(F)
	Expected *β* value	SD	*β* distribution						
(Constant)	1.92	0.28		6.85	0.000		0.31	22.25***	0.000
User need	0.15	0.10	0.17	1.59	0.11				
Perceived usefulness	0.21	0.09	0.25	2.25*	0.03				
Perceived ease of use	0.14	0.10	0.15	1.41	0.16				
Usage attitude	0.05	0.09	0.04	0.53	0.60				

*p < 0.05; **p < 0.01; ***p < 0.001

## Discussion

Of the participants, 97.94% were female; 50.52% were ≤ 30 years old; and 35.57% and 21.13% had worked in clinical services for ≥ 10 and 1–3 years, respectively. These results are nearly identical to the demographic information and seniority distributions of acute care nurses in survey studies conducted worldwide [8, 15, 18]. According to a statistical report by the Taiwan Union of Nurses Association, of the approximately 180,000 nurses registered in Taiwan by the end of January 2021, 46.9% and 41.3% possessed bachelor and junior college degrees, respectively. A high percentage of the participants in the present study had completed higher education programs, with 54.64% of them having graduated from a 2-year university technical program. Furthermore, 90.21% and 7.22% of the participants had administered CPR 1–2 times per year and 1–2 times per month, respectively, while on duty. This is consistent with the findings reported by Li and Wang [8], and Li et al. [15].

On the self-assessed emergency care competency scale, the participants scored the highest on first-aid assessment and initial treatment. Accordingly, GBL proved the participants’ abilities to assess emergency situations and select appropriate initial treatment methods, reflecting their critical thinking and problem-solving skills. This finding is consistent with the findings reported by Hsu et al. [21] and Chen and Tseng [13]. By contrast, the participants obtained the lowest average scores on arrhythmia assessment and treatment, which is consistent with the findings of Hsieh et al. [12] in their survey on the self-assessed emergency care competencies of nurses who received ACLS training at a medical center.

According to a study conducted by Tubaishat and Tawalbeh [22] regarding the difficulties encountered by nursing students in arrhythmia assessment training, nurses have the lowest confidence in and highest anxiety about accurately assessing arrhythmia when providing emergency care. However, following the game-based emergency care training program, the participants in our study had a mean score of 4.18 in the “Arrhythmia assessment and treatment” subdimension, showing that they felt secure in dealing with arrhythmia. This positive effect may have been yielded by the software product used, and the sense of security was likely built or strengthened during GBL. Therefore, on-the-job training should emphasize arrhythmia assessment.

On the usage attitude scale, the participants scored higher on the cognitive component than on the affective component, indicating that GBL related to their emergency care competencies. Lin et al. [23] reported that multimedia interactive e-book teaching, situated learning, and drawing mind maps effectively increased the accuracy of arrhythmia assessment and treatment, boosting medical professionals’ confidence.

Accordingly, GBL effectively improved the nurses’ professional competencies [12, 17, 22], consistent with the findings of Li et al. [15] and Gallegos et al. [17]. Perceived usefulness refers to users’ perceptions of a learning aid as being useful for improving their self-perceived competence and confidence and preventing them from committing mistakes at work. Perceived ease of use reflects users’ perceptions that a learning aid motivates them to learn, has a clear interface, and is easy to use.

In this study, perceived usefulness was identified as the factor most strongly affecting the participants’ emergency care competencies. GBL substantially increased the nurses’ confidence in maintaining the respiratory tract and circulation, teamwork, and CPR. The nurses’ received positive feedback from the game software, such as their perceived improvement in their emergency care competencies and the willingness of the participants to use the software again because of its easy-to-use interface and smooth game operation [4, 5, 9, 10, 16], which boosted their job performance and confidence.

This study had some limitations. First, because the participants completed the self-reported questionnaires independently, the results indicated that their perceptions of their own emergency care competencies and may not have reflected their actual competencies. Second, the emergency care competency scale on the questionnaire should be reconstructed, and its reliability and validity should be reassessed. Finally, this study did not include pretest data or a control group, and thus the interpretability of the results of using GBL software, an innovative and promising educational tool was limited.

## Conclusions

The usefulness of the game-based emergency care training program as perceived by the participants was the most crucial factor affecting the participants’ emergency care competencies out of TAM in particular, aside from other crucial factors affecting competency development. Therefore, health-care institutions should provide game-based emergency care training software to enable nurses to practice their skills repeatedly during and after on-the-job training in clinical medical education, thereby increasing their emergency care competencies in clinical practice settings. Emergency care training should be retained in nursing education and routinely offered as part of on-the-job training programs with the aid of game-based software to improve nurses’ knowledge of and attitudes toward CPR and, in turn, the quality of patient care. Game-based emergency care training reinforces nurses’ knowledge and skills, reduces their anxiety in assessing and treating arrhythmia, and strengthens their problem-solving abilities, thereby enhancing their performance and professional confidence in providing emergency care.

## Data Availability

The data presented in this study are available on request from the.
